# A study of long-term complications associated with enteral ostomy and their contributory factors

**DOI:** 10.1186/s13104-016-2304-z

**Published:** 2016-12-05

**Authors:** Umesh Jayarajah, Asuramuni M. P. Samarasekara, Dharmabandhu N. Samarasekera

**Affiliations:** Department of Surgery, Faculty of Medicine, University of Colombo, Kynsey Road, P.O. Box 271, Colombo 8, Sri Lanka

**Keywords:** Enteral ostomy, Complications, Contributory factors, Stoma

## Abstract

**Background:**

Complications of ostomy significantly affect the quality of life of ostomates. There is little evidence on the rate of long-term complications in ostomates, especially from the developing countries which include Sri Lanka. This study was aimed to describe the long-term complications of enteral ostomies and their contributory factors.

**Methods:**

A retrospective analysis was carried out on 192 patients who underwent ostomy creation over a period of 5 years. Data on type of complications, age, sex, type of ostomy, type of surgery and perioperative care by enteric stoma therapist were gathered. Associations were established using Chi square test and multiple logistic regression.

**Results:**

Out of 192 patients, only 146 patients presented regularly for follow up. The mean follow up duration was 28 months (range: 3–183). Around 34.2% developed surgical long-term complications related to the ostomy. Common complications were prolapse (n = 24, 16.4%), skin excoriation (n = 22, 15.1%) and parastomal hernia (n = 14, 9.6%). Overall complication rate was significantly less in loop ostomies (p < 0.05) and defunctioning ostomies (p < 0.05). Skin excoriation was significantly high in males (p < 0.05) and in ileostomies (p < 0.001). Parastomal hernia was commoner in end ostomies (p < 0.05). Perioperative care by enteric stoma therapist reduced the overall and specific complications (p < 0.001).

**Conclusion:**

The overall complication rate in our cohort of patients was 34.2%. The perioperative care of a stoma therapist may be very effective in preventing complications particularly in a setting with limited resources.

**Electronic supplementary material:**

The online version of this article (doi:10.1186/s13104-016-2304-z) contains supplementary material, which is available to authorized users.

## Background

The number of stoma creation surgeries are on the rise due to increase in the incidence of colorectal tumours [1] and inflammatory bowel diseases [2] which are the two most leading causes for ostomy creation [3]. Formation of a stoma results in complications which can be categorised as surgical, psychological and social complications. High complication rates have been reported in the literature which have ranged from 21 to 70 percent [4]. The long-term complication rates in colostomies can be as high as 58% [5] and in ileostomies up to 76% [6]. The common long-term complications reported are skin problems, parastomal hernia, prolapse and stenosis [7].

An essential goal in the management of ostomy patients is to maintain a high quality of life. Quality of life in ostomy patients depends on various factors. Preventing and treating long-term complications in ostomy patients is also a well-known factor that influences quality of life [8]. Knowledge of contributory factors for surgical stomal complications will help to identify those who are at a greater risk. Furthermore, timely interventions are possible with early detection so that conservative measures can be effectively implemented [9].

Management of complications requires frequent clinic visits, hospital admissions and later on surgery which has an impact on the health care expenditure especially in a low resource setting. All our patients receive their stoma appliances for free. However, they are designed to cater for uncomplicated stomas. Special appliances may be required for optimal management of complicated stomas which are not available in the government sector. Thus, it causes increased economic burden for our patients from the low income group. Therefore, preventing complications will be economically beneficial to both patients and the health care sector.

It has been shown in the literature that stoma complications can be significantly reduced by perioperative assessment and immediate care by a specialized enteral stoma therapist [10]. However, in Sri Lanka specialised enteral stoma therapists are found only in a few selective specialised surgical units. Therefore, this study was aimed to analyse the influence of perioperative care by a stoma therapist on the long-term complications.

Data on long-term complications of enteral stoma and their contributory factors in Sri Lankan population are scant. Furthermore, contributory factors for stoma related complications have not been studied in this institute. Therefore, this study was aimed to gain further insight on the possible contributory factors for long-term complication related to ostomy in a group of patients managed by us or referred to our unit and also, to identify any factors that might help to reduce long-term stoma complications.

## Methods

One hundred and ninety-two patients who were followed up at the Professorial Surgical Unit at the National Hospital of Sri Lanka from 2011 to 2015 were identified. A follow up period of minimum 3 months was considered as long-term according to previously published data [11]. Therefore, those who were followed up for a minimum of 3 months were considered for this study. Those who were not regularly followed up or those who had their stoma closed were excluded from this study. After exclusion, a sample of 146 were considered suitable for the study. Data were collected from the patient registry which was maintained prospectively by the enteral stoma therapist. Also, information was traced from clinic records and bed head tickets. Details regarding demographic factors, indications for stoma creation, type and configuration of stoma and complications were identified. All surgeries were performed by consultants or surgical registrars or senior registrars under supervision of a consultant. The long-term surgical complications of stoma considered in this study were skin excoriation, parastomal hernia, prolapse, retraction, stenosis, mucosal hyperplasia and disease recurrence. All complications were diagnosed clinically by a medical officer.

Data were analysed using SPSS 17.0 statistical software (SPSS Inc., USA). Continuous variables were expressed using mean (standard deviations). Univariate analysis was used to find associations of overall complication rate, while both univariate and multivariate analyses were done to find associations of individual complications. In the univariate analysis of associated factors, Chi square test was used to compare categorical variables. Associations were expressed in terms of odds ratio and confidence interval at 95%. In the multivariate analysis, multiple logistic regression analysis was performed and associations were expressed in terms of odds ratio and confidence interval at 95%. All statistical testing was performed at the 0.05 significance level.

## Results

The median age of the study sample analysed was 45 years (range 8–85). The mean follow up duration was 28.02 months (range 3–183). 56% (n = 82) were males. The majority of the patients had a colostomy (71.7%) and of them, the majority had a loop configuration. 44% underwent reversal subsequently (Table 1).

**Table 1:** Demographic characteristics

	Number	Percentage (%)
Sex		
Male	82	56.2
Female	64	43.8
Age (years)		
Less than 60	100	68.5
More than 60	46	31.5
Type of ostomy		
Ileostomy	41	28.1
Colostomy	105	71.9
Configuration		
Loop colostomy	80	54.8
Loop ileostomy	24	16.4
End colostomy	25	17.1
End ileostomy	17	11.6
Reversal		
Yes	60	44.1
No	76	55.9

The common indications for the ostomy were rectal and anal malignancies (28.8%) and inflammatory bowel disease (12.3%). Other indications were benign anal conditions, colonic malignancies, trauma, bowel obstruction, incontinence due to obstetric sphincter damage and polyposis syndromes. Commonest benign anal condition was fistula-in-ano (n = 13). Polyposis syndromes included familial adenomatous polyposis (n = 4), juvenile polyposis (n = 1) and Cronkhite-Canada syndrome (n = 1) (Table 2).

**Table 2:** Indications for ostomy

	Number	Percentage (%)
CA rectum and anus	42	28.8
Benign anal conditions	15	10.3
Ulcerative colitis (IBD)	12	8.2
Trauma	11	7.5
CA colon	7	4.8
Bowel gangrene/obstruction	7	4.8
Sphincter damage during labour	6	4.1
Polyposis syndromes	6	4.1
FAP + carcinoma	6	4.1
Crohn’s (IBD)	6	4.1
Surgical damage to sphincters/bowel	5	3.4
Congenital	4	2.7
Bed sore	4	2.7
Paraplegia	3	2.1
Enterocutaneous fistula	2	1.4
Anal stenosis	2	1.4
CA bladder	1	0.7
Other	7	4.8

### Types of surgery

Surgical procedures that led to an ostomy included, abdominoperineal resection, anterior resection, total colectomy and Hartmann’s procedure. Defunctioning colostomy was the commonest ostomy procedure (54.2%) (Table 3).

**Table 3:** Surgical procedures that lead to an ostomy

	Number	Percentage (%)
Total colectomy and end ileostomy	14	9.6
Defunctioning traverse loop colostomy	36	24.7
Defunctioning sigmoid colostomy	43	29.5
Abdominoperineal resection (APR)	19	13.0
Anterior resection (AR)	10	6.8
Ileal pouch anal anastomosis (IPAA) with a diverting ileostomy	9	6.2
Defunctioning ileostomy	8	5.5
Hartmann’s procedure	5	3.4
Left hemicolectomy and transverse loop colostomy	1	0.7
Subtotal proctocolectomy	1	0.7

### Long-term surgical complications of enteral ostomy

Around 34.2% developed long-term complications related to ostomy (ileostomy 36.6%, colostomy 33.3%), of which 21.9% developed one complication while 11.7% developed 2 or more complications. Common complications were prolapse (n = 24, 16.4%), skin excoriation (n = 22, 15.1%) and parastomal hernia (n = 14, 9.6%). Other complications were patchy hyperplasia of the mucosa (n = 3, 2.1%), retraction (n = 2, 1.4%), stenosis (n = 1, 0.7%), recurrence (n = 2, 1.4%) and stoma blockage (n = 2, 1.4%).

### Contributory factors for ostomy complications

The highest rate of complications were noted in end ileostomies (n = 9, 52.9%) followed by end colostomies (n = 10, 40.0%), while lowest complication rates were seen in loop ileostomies (n = 6, 25%) followed by loop colostomies (n = 25, 31.2%). Loop ostomies were associated with significantly less complications than end ostomies (odds ratio = 0.437, 95% CI 0.206–0.924, p = 0.028). Defunctioning colostomies had significantly low complication rates compared to other surgeries (odds ratio = 0.437, 95% CI 0.217–0.882, p = 0.020). Higher complication rates were seen in younger age groups (age less than 60) and in males, though it was not statistically significant (p = 0.063, p = 0.161 respectively).

Stomal prolapse was significantly low in those who were diagnosed with malignancies (odds ratio = 0.330, 95% CI 0.106–1.027, p = 0.047). Lack of perioperative assessment by enteral stoma therapist had significantly higher rates of prolapse. (odds ratio = 4.176, 95% CI 1.550–11.257, p = 0.003). Furthermore, both above mentioned factors were the only significant predictors of stomal prolapse in the multivariate analysis (Table 4). Stoma prolapse was commonest in loop colostomies (20%) followed by end ileostomies (17.6%). Lowest rate of prolapse was seen in loop ileostomies (8.3%) followed by end colostomies (12%), though it was not statistically significant. There was no significant association with age, sex and the type of surgery.

**Table 4:** Multivariate analysis of contributory factors for stoma prolapse

	Odds ratio	95% CI for odds ratio		p value
		Lower	Upper	
Lack of perioperative care by stoma therapist	5.230	1.851	14.774	0.002
Males	1.366	0.520	3.591	0.527
Age	0.994	0.966	1.022	0.665
Malignancies	0.252	0.069	0.915	0.036
Constant	7.911	–	–	0.042

In the univariate analysis, skin excoriation was seen at a significantly higher rate in ileostomies compared to colostomies (odds ratio = 6.287, 95% CI 2.389–16.547, p = 0.0001). Perioperative assessment by stoma therapist did not reduce the incidence of skin excoriation significantly (odds ratio = 1.754, 95% CI 0.699–4.404, p = 0.228). The number of skin excoriation was higher in males, though it was not statistically significant. (p = 0.089). There was no significant association with age, diagnosis and the type of surgery.

In the multivariate analysis, a significant association was found with ileostomies compared to colostomies and in males compared to females. The influence of perioperative assessment by stoma therapist became statistically significant (Table 5).

**Table 5:** Multivariate analysis of contributory factors for skin excoriation

	Odds ratio	95% CI for odds ratio		p value
		Lower	Upper	
Lack of perioperative care by stoma therapist	3.924	1.188	12.960	0.025
Males	4.210	1.219	14.539	0.023
Age	0.975	0.943	1.008	0.132
Ileostomies	13.097	3.549	48.332	0.000
End configuration	1.553	0.517	4.668	0.433
Constant	0.972	–	–	0.974

Parastomal hernia was seen significantly commoner in females (odds ratio = 3.845, 95% CI 1.853–7.976, p = 0.0001). The proportion of parastomal hernia was significantly higher in those who were not assessed peri operatively by a stoma therapist (odds ratio = 7.895, 95% CI 1.699–36.681, p = 0.002). Parastomal hernia was significantly high in end colostomies (28%, odds ratio = 6.333, 95% CI 1.986–20.195, p = 0.001).

In the multivariate analysis, association with perioperative care by stoma therapist and type of ostomy were statistically significant in the logistic regression model (Table 6). Further details of complications of ostomy is available in the data file that is attached (Additional file 1).

**Table 6:** Multivariate analysis of contributory factors for parastomal hernia

	Odds ratio	95% CI		p value
		Lower	Upper	
Lack of perioperative care by stoma therapist	7.095	1.444	34.872	0.016
Females	3.463	0.925	12.969	0.065
Age	1.016	0.977	1.056	0.438
End colostomies	7.972	1.941	32.740	0.004
Constant	0.307	–	–	0.326

## Discussion

The literature on ostomy complications show an overall complication rate ranging from 21 to 70% [4]. The complication rate in our study was 34.7% with the majority having only one complication. Furthermore, it has been mentioned that the majority of stoma complications occur early which is within two weeks after discharge [12]. The data on long-term complications excluding acute complications is limited and the majority of the studies have focussed on early complications. A retrospective analysis done on long-term complications in defunctioning ostomies reported an overall complication rate of 60% which is a comparatively higher rate [11]. Another study by Duchesne et al. [10] reported that 25% had stoma complications out of which 39% occurred within the first month, which indicates only 15.25% of complications occurred after 1 month. The reason for this wide range may be due to the difference in follow up duration, and the differences in proportions of different types of ostomies, diagnosis and the type of surgery. Since the complication rate is influenced by multiple risk factors, it has been difficult to analyse the contributory factors accurately.

In our study, the lowest complication rates were seen in loop ileostomies (25%) followed by loop colostomies (31.2%). Furthermore, end ostomies had a higher rate of complications than loop ostomies. A retrospective analysis done in 2007 reported the lowest complication rates for end colostomies [11]. However, a systematic review consisting seven studies which included 3 randomised clinical studies [13] and a retrospective study [14] that specifically compared complications and outcome in ileostomies and colostomies, indicated that lowest complication rates were seen for loop ileostomies. Therefore, the result of our study were comparable with those studies. Defunctioning ostomies had a significantly low rate of complications compared to other types of surgeries. This may be because, defunctioning ostomies were loop ostomies and the surgery was less complicated compared to other surgeries which involved greater abdominal access and more bowel handling.

The common complications seen in this study were prolapse, skin excoriation and parastomal hernia which are comparable with other similar studies [3, 11, 15]. However, the reported rates of these complications in these studies were different. This may be because of the difference in the nature of the stoma, types or surgeries, duration of follow up and demographic factors in these studies.

We noted a significant reduction in the overall complication rate when perioperative care was provided by a specialised enteral stoma therapist which is an important finding. In the majority of the studies, it has been shown that the influence of stoma therapist had a positive effect on the outcome which included complications due to surgery as well [10]. Increase in the rate of complications gives rise to additional economic burden to both patients and the health sector. Furthermore, reduction of complications has a positive effect on the quality of life of the patients. Therefore, incorporating a specialised stoma therapist may be an effective preventive measure to reduce the complication rates in a developing country like Sri Lanka where the resources are limited.

In our study, analysis of parastomal hernias revealed a significant association in females. Other studies on parastomal hernia related to enteral ostomy did not give a significant association in females [16, 17]. However, in a retrospective analysis of ileal conduits, parastomal hernias were shown to be significantly high in females [18]. Furthermore, this study also showed that perioperative care by stoma therapist significantly reduced the rate of parastomal hernias. Also, parastomal hernia was commonest in end colostomies which is in agreement with similar studies [19].

Our study showed that skin excoriation was significantly commoner in ileostomies. This observation is in agreement with consensus throughout the literature [20]. It is suggested that it is common because small intestinal output is frequent and more irritant due to its caustic nature [21]. Even though our study showed an association in males, a similar retrospective study on skin problems of ostomy did not show an association between skin excoriation and gender [20].

In our study, the rate of prolapse was common among loop colostomies which is in agreement with recent publications. A review of literature of over 50 years showed significantly higher incidence of prolapse in loop colostomies compared to loop ileostomies [22]. Prolapse was significantly low in those who were diagnosed with malignancies. This may be because, in treating malignancies end colostomies which has the lowest rate of prolapse, is commonly used in the treatment.

It is important to note that a greater proportion of complications related to enteral ostomies may be preventable. Presence of complications require frequent changes of appliances, use of special appliances and frequent clinic visits and hospital admissions which pose a significantly higher economic burden in a low resource setting. Therefore, prevention of complications is key in a low resource setting as ours. An effective way of prevention of stoma related complications is proper pre-operative siting. Improper pre-operative siting can give rise to skin irritation, leakage and poor visualisation of stoma, leading to psychological distress and poor post-operative adjustment [23, 24]. Therefore, proper siting should be given great priority, even if it is an emergency setting and involvement of a specialised stoma nurse in pre-operative care should always be considered [25], particularly in patients who are obese or those with unfavourable body habitus [26].

Another effective way to minimise complications is by pre-operative education and counselling regarding post-operative expectations [25]. Therefore involvement of a stoma care nurse would be ideal, particularly in a low resource setting. Pre-operative and early post-operative training in handling stomas is a useful method to prevent early complications such as skin irritation [27]. These will help patient to identify stoma related complications early so that conservative management plans may be implemented effectively and surgical interventions can be minimised. Furthermore, patients who are at a greater risk of stoma related complications should be followed up more frequently to identify the complications early.

Our study is limited by its retrospective nature. The other contributory factors of complications such as stenosis, retraction etc. could not be evaluated as the sample is not powered enough to detect the significance. Thus a prospective study with a larger sample size would have been ideal.

## Conclusions

The overall long-term surgical complication rate in our unit was 34.2%. The common complications were prolapse, skin excoriation and parastomal hernia. The important observation of this study is that both overall and specific complications were reduced in those who were peri operatively assessed by a specialised enteral stoma therapist. Therefore, the care of a stoma therapist may be very effective in preventing stoma complications, especially in a poor resource setting like Sri Lanka.

Furthermore, special attention may be given to those who are having risk factors of complications by means of frequent follow up assessments, education and training to prevent such complications.

## Additional file

**Additional file 1.** Details of complications of ostomy–data file.

## References

[1] de Paula MA (1996). Performance of stoma therapy in the process of rehabilitation of ostomy patients. Revista brasileira de enfermagem.

[2] Qin X (2011). What caused the recent worldwide increase of inflammatory bowel disease: should sucralose be added as a suspect?. Inflamm Bowel Dis.

[3] Krouse RS, Grant M, Rawl SM, Mohler MJ, Baldwin CM, Coons SJ, McCorkle R, Schmidt CM, Ko CY (2009). Coping and acceptance: the greatest challenge for veterans with intestinal stomas. J Psychosom Res.

[4] Shabbir J, Britton DC (2010). Stoma complications: a literature overview. Colorectal Dis.

[5] Londono-Schimmer EE, Leong APK, Phillips RKS (1994). Life Table analysis of stomal complications following colostomy. Dis Colon Rectum.

[6] Leong APK, Londono-Schimmer EE, Phillips RKS (1994). Life-table analysis of stomal complications following ileostomy. Br J Surg.

[7] Harris DA, Egbeare D, Jones S, Benjamin H, Woodward A, Foster ME (2005). Complications and mortality following stoma formation. Ann Royal Coll Surg Engl.

[8] Pittman J, Rawl SM, Schmidt CM, Grant M, Ko CY, Wendel C, Krouse RS (2008). Demographic and clinical factors related to ostomy complications and quality of life in veterans with an ostomy. J Wound Ostomy Cont Nurs.

[9] Buchmann P, Huber M (2007). The complicated stoma–late complications, conservative and surgical management. Therapeutische Umschau Revue Therapeutique.

[10] Duchesne JC, Wang YZ, Weintraub SL, Boyle M, Hunt JP (2002). Stoma complications: a multivariate analysis. Am Surg.

[11] Caricato M, Ausania F, Ripetti V, Bartolozzi F, Campoli G, Coppola R (2007). Retrospective analysis of long-term defunctioning stoma complications after colorectal surgery. Colorectal Dis.

[12] Persson E, Berndtsson I, Carlsson E, Hallen AM, Lindholm E (2010). Stoma-related complications and stoma size—a 2-year follow up. Colorectal Dis.

[13] Tilney HS, Sains PS, Lovegrove RE, Reese GE, Heriot AG, Tekkis PP (2007). Comparison of outcomes following ileostomy versus colostomy for defunctioning colorectal anastomoses. World J Surg.

[14] Rullier E, Le Toux N, Laurent C, Garrelon JL, Parneix M, Saric J (2001). Loop ileostomy versus loop colostomy for defunctioning low anastomoses during rectal cancer surgery. World J Surg.

[15] Salvadalena G (2008). Incidence of complications of the stoma and peristomal skin among individuals with colostomy, ileostomy, and urostomy: a systematic review. J Wound Ostomy Cont Nurs.

[16] Ripoche J, Basurko C, Fabbro-Perray P, Prudhomme M (2011). Parastomal hernia. A study of the French federation of ostomy patients. J Visc surg.

[17] Pilgrim CH, McIntyre R, Bailey M (2010). Prospective audit of parastomal hernia: prevalence and associated comorbidities. Dis Colon Rectum.

[18] Donahue TF, Bochner BH, Sfakianos JP, Kent M, Bernstein M, Hilton WM, Cha EK, Yee AM, Dalbagni G, Vargas HA (2014). Risk factors for the development of parastomal hernia after radical cystectomy. J Urol.

[19] Hiranyakas A, Ho YH (2010). Laparoscopic parastomal hernia repair. Dis Colon Rectum.

[20] Nybæk H, Knudsen DB, Laursen TN, Karlsmark T, Jemec GBE (2009). Skin problems in ostomy patients: a case-control study of risk factors. Acta dermato-Venereologica.

[21] Hellman J, Lago CP (1990). Dermatologic complications in colostomy and ileostomy patients. Int J Dermatol.

[22] Hanna MH, Vinci A, Pigazzi A (2015). Diverting ileostomy in colorectal surgery: when is it necessary?. Langenbeck’s archives of surgery/Deutsche Gesellschaft fur Chirurgie.

[23] Mäkelä J, Niskasaari M (2006). Stoma care problems after stoma surgery in Northern Finland. Scand J Surg.

[24] Robertson I, Leung E, Hughes D, Spiers M, Donnelly L, Mackenzie I, Macdonald A (2005). Prospective analysis of stoma-related complications. Colorectal Dis.

[25] Aukett LK, Bonsaint R, Corbin C, Daum F, Ebbert J, Farah L, Farrar J, Fleshman J, Gabrielson L, Gray M, Grindel C (1994). National guidelines for enterostomal patient education. Dis Colon Rectum.

[26] Colwell JC, Fichera A (2005). Care of the obese patient with an ostomy. J Wound Ostomy Cont Nurs.

[27] Bass E, Del Pino A, Tan A, Pearl R, Orsay C, Abcarian H (1997). Does preoperative stoma marking and education by the enterostomal therapist affect outcome?. Dis Colon Rectum.

